# Maintaining the structural integrity of the *bamboo mosaic virus* 3′ untranslated region is necessary for retaining the catalytic constant for minus-strand RNA synthesis

**DOI:** 10.1186/1743-422X-10-208

**Published:** 2013-06-24

**Authors:** I-Hsuan Chen, Chiu-Heiu Chu, Jen-Wen Lin, Yau-Heiu Hsu, Ching-Hsiu Tsai

**Affiliations:** 1 Graduate Institute of Biotechnology, National Chung Hsing University, Taichung 40227, Taiwan; 2 Graduate Institute of Medical Laboratory Science and Biotechnology, China Medical University, Taichung 404, Taiwan

## Abstract

**Background:**

*Bamboo mosaic virus* (BaMV) and the *Potato virus X* (PVX) are members of the genus *Potexvirus* and have a single-stranded positive-sense RNA genome. The 3′-untranslated region (UTR) of the BaMV RNA genome was mapped structurally into ABC (a cloverleaf-like), D (a stem-loop), and E (pseudoknot) domains. The BaMV replicase complex that was isolated from the infected plants was able to recognize the 3′ UTR of PVX RNA to initiate minus-strand RNA synthesis *in vitro*.

**Results:**

To investigate whether the 3′ UTR of PVX RNA is also compatible with BaMV replicase *in vivo*, we constructed chimera mutants using a BaMV backbone containing the PVX 3′ UTR, which was inserted in or used to replace the various domains in the 3′ UTR of BaMV. None of the mutants, except for the mutant with the PVX 3′ UTR inserted upstream of the BaMV 3′ UTR, exhibited a detectable accumulation of viral RNA in *Nicotiana benthamiana* plants. The *in vitro* BaMV RdRp replication assay demonstrated that the RNA products were generated by the short RNA transcripts, which were derived from the chimera mutants to various extents. Furthermore, the V_max_/K_M_ of the BaMV 3′ UTR (rABCDE) was approximately three fold higher than rABCP, rP, and rDE in minus-strand RNA synthesis. These mutants failed to accumulate viral products in protoplasts and plants, but were adequately replicated *in vitro*.

**Conclusions:**

Among the various studied BaMV/PVX chimera mutants, the BaMV-S/PABCDE that contained non-interrupted BaMV 3′ UTR was the only mutant that exhibited a wild-type level of viral product accumulation in protoplasts and plants. These results indicate that the continuity of the domains in the 3′ UTR of BaMV RNA was not interrupted and the domains were not replaced with the 3′ UTR of PVX RNA *in vivo*.

## Background

The *Bamboo mosaic virus* (BaMV) has a single-stranded positive-sense RNA genome that is 6366 nt in length and contains a 5′ m^7^GpppG cap and a 3′ poly (A) tail [1]. Two major subgenomic RNAs 2 and 1 kb in length share a 3′ co-terminus with genomic RNA. The open reading frames 1 to 5 encode polypeptides 155, 28, 13, 6, and 25 kDa in size. The 155-kDa polypeptide has been divided into the following three functional domains: the capping enzyme domain [2,3], the helicase-like domain with a RNA 5′-triphosphotase activity [4], and the RNA-dependent RNA polymerase (RdRp) domain [5]. The RdRp domain interacted specifically with the 3′ untranslated region (UTR) of BaMV genomic RNA *in vitro*[6].

Viral-encoded RdRp is involved in minus- and plus-strand viral RNA syntheses. These two processes are usually asymmetric, leading to a 20- to 100-fold excess of positive-strand over minus-strand RNA [7]. In general, the viral-encoded RdRp must interact specifically with the 3′ UTR of the positive-sense RNA to initiate minus-strand RNA synthesis. The sequences or structures in the 3′ end, including tRNA-like structure (TLS), a poly (A) tail, a stem-loop structure, and a non-TLS heteropolymeric sequence, present the specificity for recognition by the RdRp complex for minus-strand RNA synthesis, such as those identified in *Turnip crinkle virus* (TCV) [8], *Alfalfa mosaic virus*[9], *Turnip yellow mosaic virus*[10], and *Brome mosaic viru* (BMV) [11].

**Figure 1:**
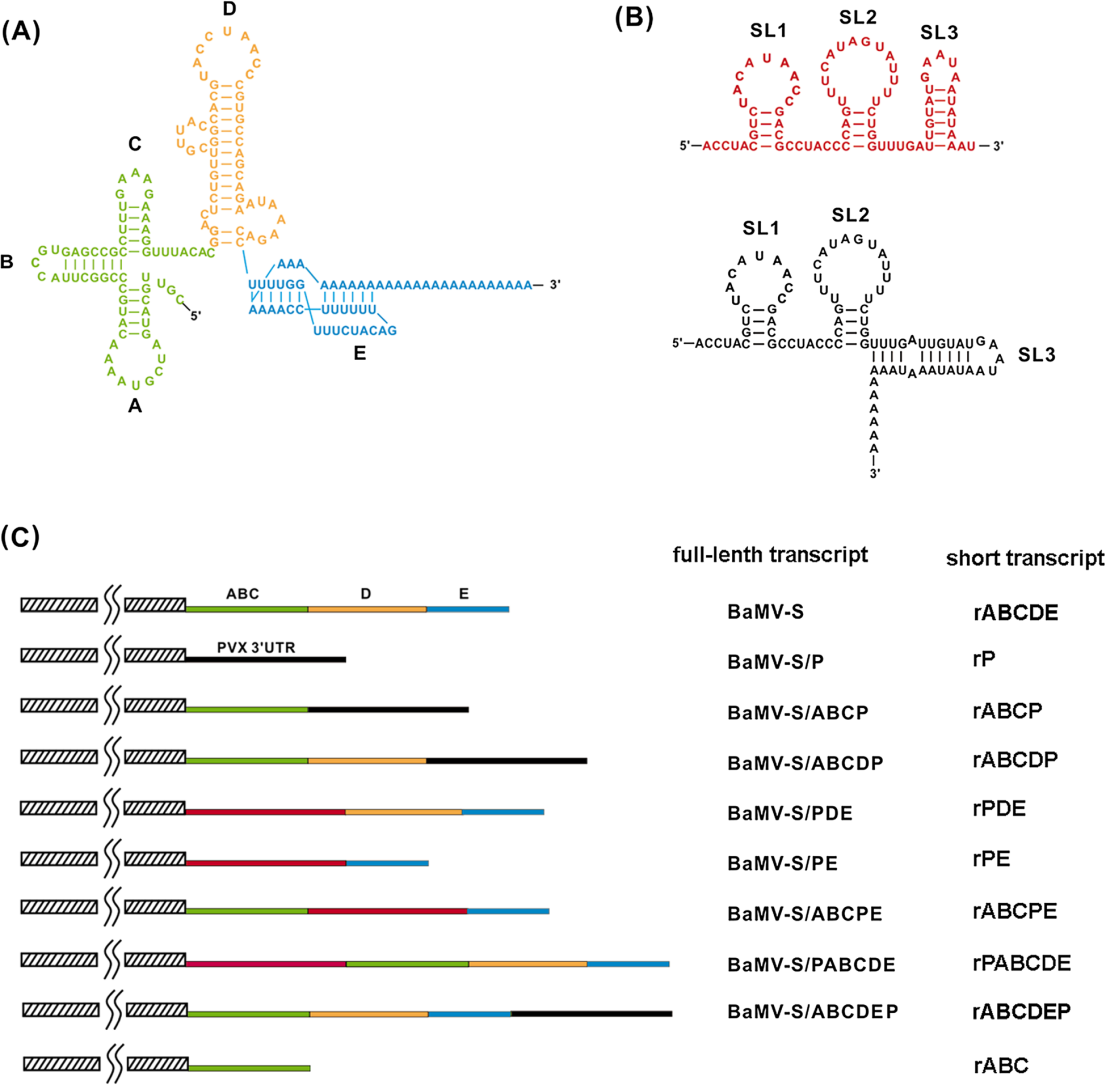
**The diagram of the 3′ UTR structures of BaMV and PVX and the various chimera constructs.** (**A**) The primary sequence and secondary structure of the BaMV-3′ UTR. BaMV-3′ UTR consists of three small stem-loops forming a clover leaf-like structure (ABC domain in green), one major stem-loop including a buldge and an internal loop (D domain in orange), and a pseudoknot (E domain in blue). (**B**) The primary sequence and two alternative secondary structure of the PVX 3′ UTR in red without poly (**A**) and in black with poly (**A**) tail [20]. Numbering of the stem-loops (SL1-3) is as indicated. (**C**) Schematic representation of the chimeric constructs based on a full-length BaMV-S cDNA clone. Hatched blocks: the coding sequence upstream of the 3′ UTR of BaMV RNA; blocks in colors indicated different domains shown in (**A**); the PVX 3′ UTR sequence in red when inserted in the 3′ UTR of BaMV, in black when fused to the very 3′-end of chimera. All constructs have a 40-adenosine-tract followed by an *EcoICR* I cleavage site at it’s 3′-end. Construct designations are shown at the right with the full-length transcript for protoplast and plant inoculation and with the short transcript for *in vitro* replication assay.

The *cis*-acting elements in the 3′ UTR of BaMV RNA for minus-strand RNA synthesis was identified in an *in vitro* replication assay by using the replicase complex that was derived from BaMV-infected *Nicotiana benthamiana*[12,13] and in electrophoretic mobility shift and footprinting assays using the recombinant polymerase domain of ORF1, which is prepared from *Escherichia coli*[6]. These elements, including the tertiary structure [14,15] and the potexviral conserved hexamer motif [16] in the 3′ UTR, are required for the efficient accumulation of viral products in protoplasts. The tertiary structure, which comprises 3 stem-loops constitutes the ABC domain (a cloverleaf-like structure), the D domain, and the E domain (a pseudoknot structure) (Figure 1) [14,17]. The ABC domain functions in both minus-strand RNA synthesis and viral long-distance movement [15]. The D domain contains the potexviral conserved sequence at the apical loop, which is required for efficient viral RNA replication, [16] and the polyadenylation signal at the internal loop [18]. The pseudoknot domain provides the initiation site for minus-strand RNA synthesis [19].

The 3′ UTR of the *Potato virus X* (PVX) RNA was predicted to form 3 stem-loops (Figure 1) [20] and may be recognized by the replication machinery to initiate minus-strand RNA synthesis. The replicase complex that was isolated from the BaMV-infected plants may also recognize the PVX 3′ UTR to initiate minus-strand RNA synthesis [12] and yield up to 36% template activity compared with that of the BaMV 3′ UTR. Conversely, the 3′ UTR of BaMV combined with PVX replicase produced approximately 63% template activity compared with that of PVX 3′ UTR [12]. Markedly lower levels of template activity were observed in non-potexviral RNA, such as the 3′ TLS of the *Cucumber mosaic virus* (CMV), with both viral replicases, indicating the involvement of specific common elements in the 3′ UTRs in the initiation of the minus-strand RNA synthesis of potexviruses. To identify these elements, mutants were created by replacing the domains in the 3′ UTR of BaMV RNA with the corresponding sequence from the 3′ UTR of PVX RNA or inserting the corresponding sequence from the 3′ UTR of PVX RNA. The chimeric RNAs were used to test the viral viability in protoplasts and plants as well as the template activity in an *in vitro* replication assay.

## Results

Various BaMV/PVX chimera mutants of the structural elements in the 3′ UTR of the BaMV and PVX were created to examine the relationship between structure and function based on a full-length infectious cDNA clone (pBS2-8) of BaMV strain S (BaMV-S) [15]. The entire BaMV 3′ UTR was replaced with the 3′ UTR of PVX RNA in the BaMV-S/P mutant. The DE, E, D, ABC, and ABCD regions of the BaMV-S were replaced with the PVX 3′ UTR to generate BaMV-S/ABCP, -/ABCDP, -/ABCPE, -/PDE, and -/PE mutants, respectively (the replacement mutants) (Figure 1). The PVX 3′ UTR was inserted in the upstream and downstream of the 3′ UTR of BaMV to create BaMV-S/PABCDE and -/ABCDEP, respectively (the insertion mutants).

### Most chimera mutants failed to produce infectious RNA *in vivo*

**Figure 2:**
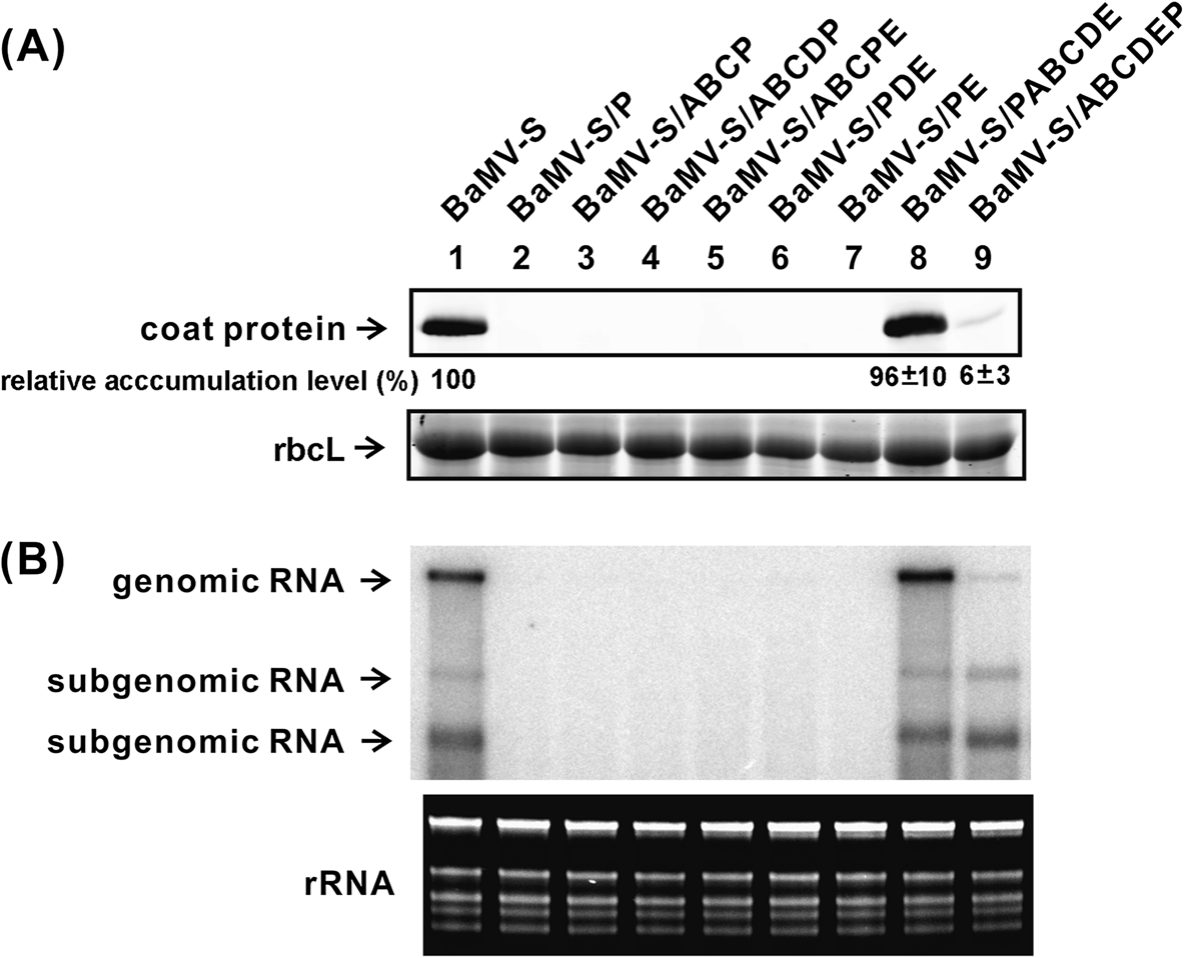
**Analysis of BaMV protein and RNA accumulation of BaMV-S and its derivatives in *N. benthamiana *protoplasts.** (**A**) Western blot analysis of BaMV coat protein (25 kDa) in protoplasts inoculated with the RNA transcripts (5 μg) indicated on the top of each lane. Total protein extracts were separated on a 12% SDS-polyacrylamide gel, blotted, and probed with a rabbit-anti BaMV coat protein antiserum, followed by a fluorescence-labeled anti-rabbit IgG antibody. The bolt was scanned and quantified afterwards. rbcL indicates the large subunit of RuBisCo stained with coomassie blue as a loading control. (**B**) Northern blot analysis of BaMV genomic (6.4 kb) and two subgenomic (2.0 and 1.0 kb) RNAs in protoplasts. The blot was probed with a ^32^P-labelled RNA complementary to a 600-bp area at the 3′-end of genomic RNA. The ribosomal RNA (rRNA) shown under the blot served as the sample loading control.

No BaMV-related RNA or protein products were detected in the protoplasts that were transfected with full-length RNA transcripts, BaMV-S/P, -/ABCP, -/ABCDP, -/PDE, -/PE, or -/ABCPE (all the replacement mutants). However, the two detectable mutants were the insertion mutants BaMV-S/PABCDE (96%) and BaMV-S/ABCDEP (6%) at 48 h post inoculation (Figure 2). Similar results were observed in plants for all the replacement mutants that were inoculated with the same set of full-length RNA transcripts. Although BaMV-S/ABCDEP could be detected in protoplasts for approximately 6%, it failed to be detected in plants. The only one mutant survived in plants is BaMV-S/PABCDE. The progeny virus was produced; however, symptom development and systemic movement were delayed by 1 d in plants compared with those inoculated with BaMV-S. The BaMV-S/PABCDE RNA was isolated from plant tissue and sequenced to confirm that no undesired mutations were introduced.

### The chimeras do not affect the translation and stability

**Figure 3:**
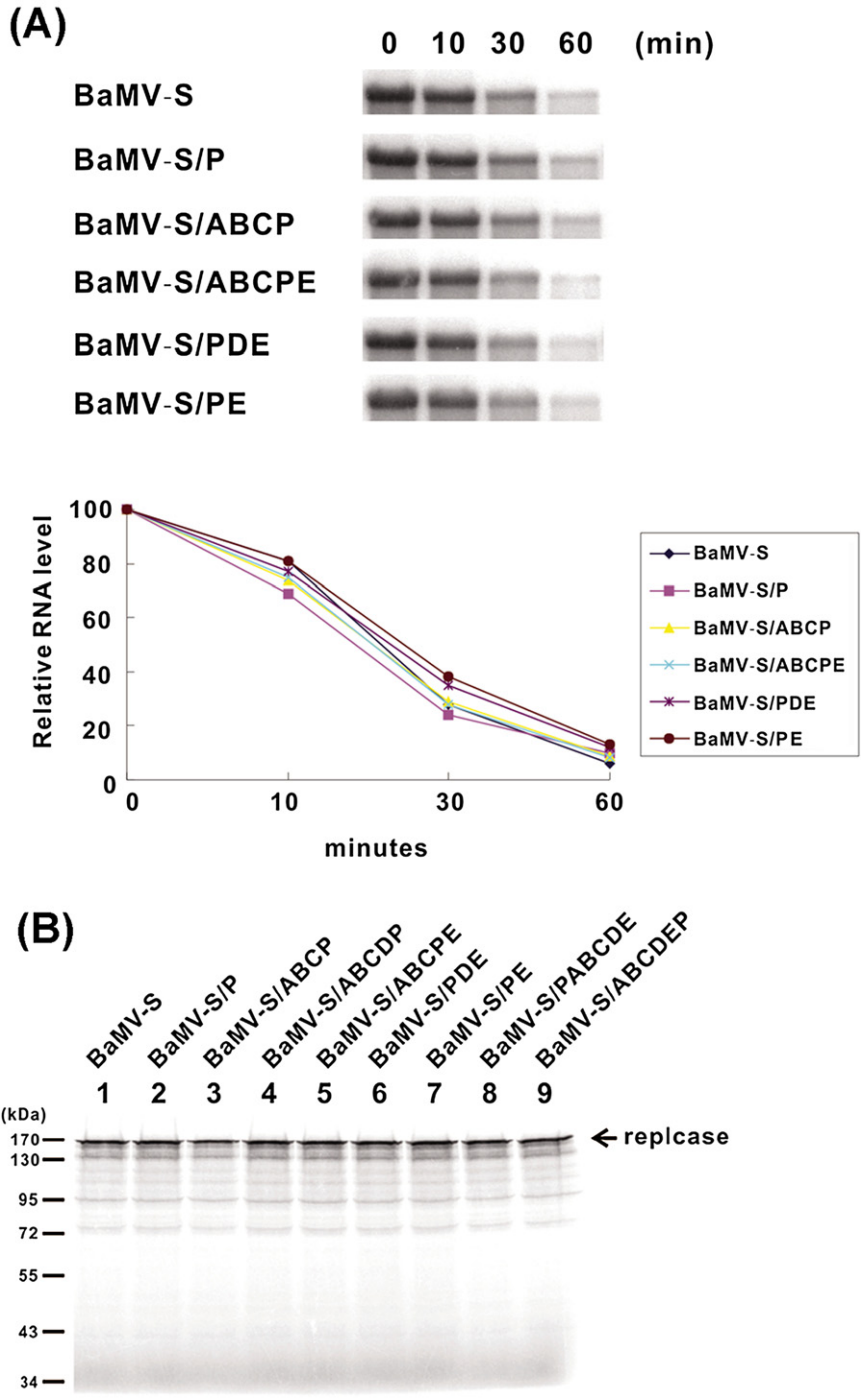
**RNA stability and *in vitro *translation assays of BaMV-S and its derivatives in wheat germ extract.** (**A**) RNA stability assay in wheat germ. RNAs were extracted from *in vitro* translation reaction at various incubation times as indicated and were subjected to a Northern blot analysis. (**B**) *In vitro* translation was performed in wheat germ extract with RNA of BaMV-S and its derivatives. The products were separated by 10% SDS-PAGE and analyzed by a phosphorimager (Fujifilm BAS 2500). The molecular masses of the markers are indicated on the left. The arrow indicates the position of the replicase.

Because the viral products of most of the chimeras were undetectable in protoplasts after inoculation, we have wondered whether the translation or the stability of these chimeras is affected. To address this question, BaMV-S, BaMV-S/P, -/ABCP, -/ABCPE, -/PDE, -/PE, and -/ABCDEP were incubated in wheat germ extract (TnT Coupled Transcription/Translation System) for various time periods and then subjected to Northern blot analysis [21]. The result showed that the RNA decay profile of these chimeras was similar to that of BaMV (Figure 3A), and suggested that the stability may not be the main cause of the chimeras failed to accumulate *in vivo*. This result was supported by *in vitro* translation assay that all constructs could translate the replicase at similar levels (Figure 3B). Therefore, we concluded that the defect of the most chimeras in plant cells is not due to their stability or translatability.

### All the chimeras can be used as the templates in the *in vitro* RdRp assay

**Figure 4:**
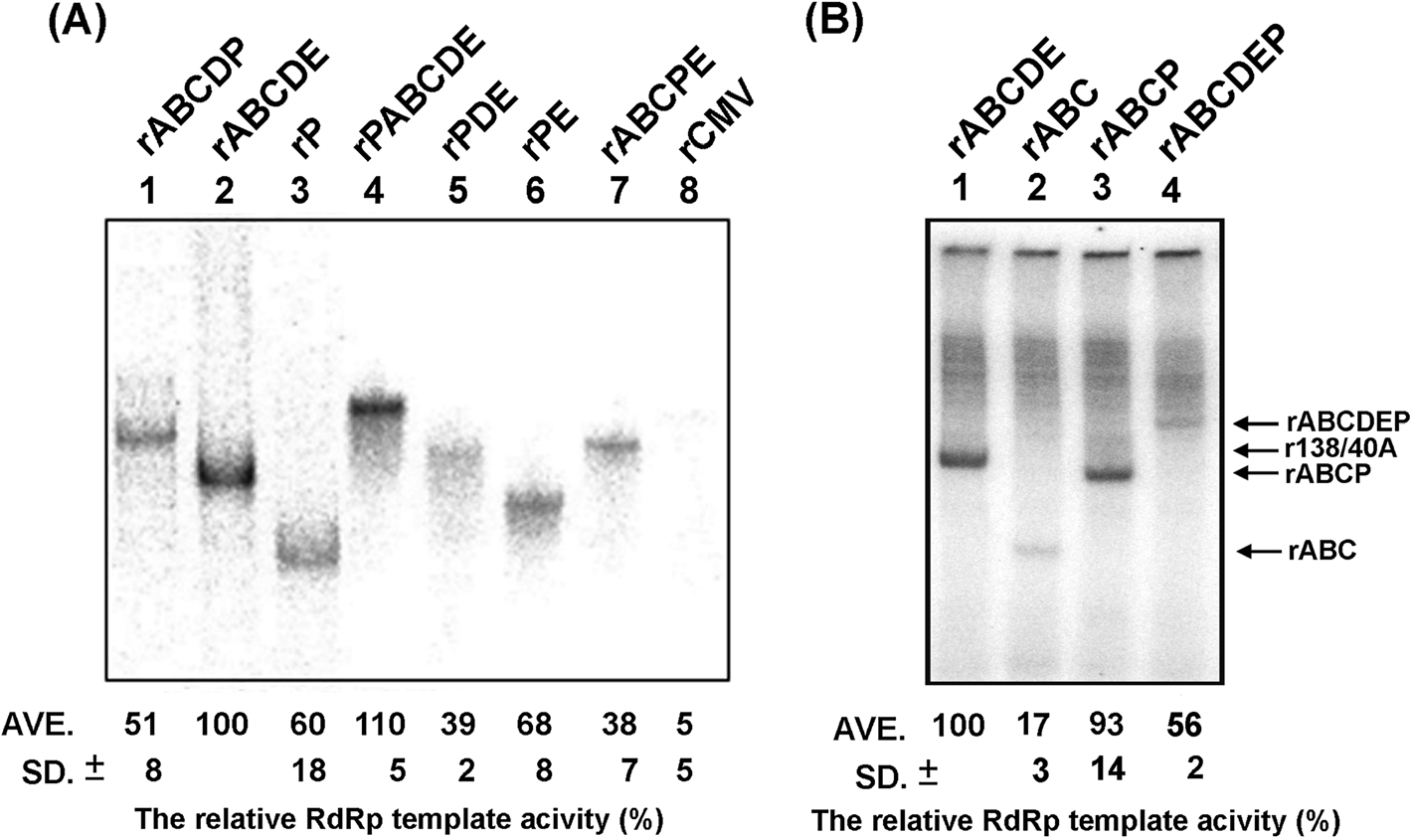
**Analysis of template activity of BaMV-3′ UTR RNA and its derivatives in an *in vitro *replication assay.** (**A**) Autoradiograph of the [α-^32^P] UTP-labeled minus-strand RNA products were separated on a 5% polyacrylamide gel, quantified with a phosphorimage system, and normalized to the amounts of rABCDE-derived products. (**B**) Same as in (**A**) with different set of samples indicated on the top of each lane.

To clarify the effects of the mutation in the 3′ UTR on replication, all the constructs were analyzed using *in vitro* BaMV RNA replication. The T7 promoter-containing PCR products were prepared to allow the generation of short transcripts, which encompass the 3′ UTR of the individual mutant constructs *in vitro*. The rABCDE transcript, which contained the 3′ UTR of BaMV RNA and 40 additional adenylate residues at the 3′ end, was used as the positive control. The RNA accumulation of the rABCDE transcript in the *in vitro* replication assay was arbitrarily assigned as 100%. The rPABCDE construct with PVX 3′ UTR located upstream of the rABCDE exhibited 110% RNA accumulation. Conversely, the rP that represents the heterologous PVX 3′ UTR displayed an approximate RNA accumulation of 60%. The addition of the homologous BaMV ABC domains to the 5′ end of the PVX 3′ UTR (rABCP) resulted in an increase in the wild-type level (Figure 4A), indicating that the ABC domain plays a vital role in BaMV RNA replication. Similarly, rABC without the RdRp binding domain (D and E) [6] could only have 17% template activity, with the addition of the PVX 3′ UTR could exhibit almost wild-type template activity (Figure 4B). The mutants with the ABC domain replaced by the 3′ UTR of PVX RNA, such as rPDE and rPE, exhibited 39% and 68% RNA accumulation, respectively. These results indicate that the 3′ UTR of PVX RNA could functionally complement the D and E domains of BaMV RNA *in vitro.* These results can be also linked to the colinearity of the helicase-like and RdRp domains of BaMV that bind to the ABC and DE domains, respectively [6,15]. However, for rABCDP and rABCPE, only 51% and 38% RNA accumulation were achieved, respectively (Figure 4), which implies that these functional homologous regions (DE vs. P) affect each other when they are fused in tandem. Although the transcript rPABCDE contains DE and P, the linearity of ABC and DE is remained to fit the continuity of helicase-like and RdRp domains. Whereas mutant rABCDEP with DE and P homologous domains connected to each other could have the interference of the RdRp binding that resulted in only 56% template activity (Figure 4B).

The construct rABCP was observed to have the wild-type level of RNA accumulation in an *in vitro* BaMV RNA replication assay; however, no viral products were detected in protoplasts or plants that were transfected with the full-length mutant transcript. To ensure that effective amounts of RNA were used to induce detectable levels of viral RNA replication in plants, the plants were transfected directly with up to 20 μg of BaMV-S/ABCP RNA or with lysates prepared from protoplasts that were transfected with the same amounts of RNA. However, no viral products were detected using Western and Northern blotting analyses. A possible explanation for this result is that the suboptimal kinetics did not allow BaMV-S/ABCP to pass over the RNA threshold and effectively initiate the virus life cycle in the host cell. Therefore, the kinetic parameters between the replicase complex and several mutant RNA templates were analyzed to determine the possible roles of the various 3′ UTR domains in BaMV RNA replication.

### Chimera rABCP has slower replication rate than that of wild-type

**Figure 5:**
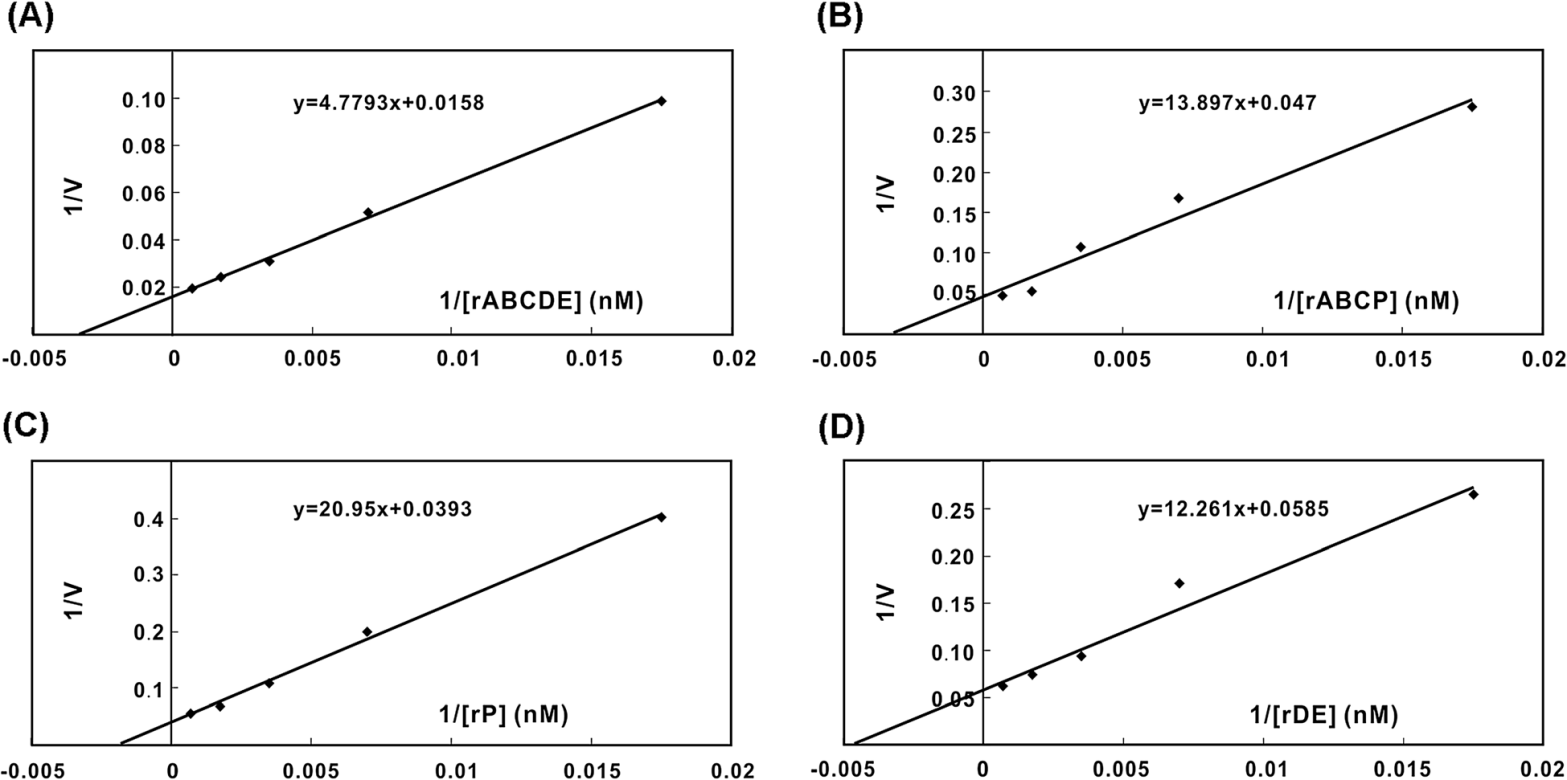
**Lineweaver-Burke analysis of BaMV RNA replication rates with the replicase complex in the presence of varying concentration of different RNA templates.** RdRp assays with the template indicated on each panel were performed as described in Methods. The concentrations of each template used were ranged from 30 to 1500 nM. All data represent the mean of at least three independent experiments.

Transcripts of rABCDE, rABCP, rDE, and rP were studied to focus on the ABC and DE domains. A Michaelis-Menten hyperbolic graph, which was based on the determined initial velocity and substrate concentration (Figure 5), was transformed into a Lineweaver-Burk plot. The K_M_ values for rABCDE, rABCP, rP, and rDE were calculated as 302, 295, 533, and 209 nM, respectively (Table 1). The similar K_M_ values of rABCDE and rABCP imply that they exhibit comparable replicase complex-binding efficiencies. However, rABCDE displayed a V_max_/K_M_ that was a threefold higher than that of rABCP, which indicates that rABCP replicated at a slower rate than rABCDE. Conversely, compared to rP (K_M_=533 nM), the addition of the BaMV ABC domain in rABCP (K_M_=295 nM) enhanced the interaction between the 3′ UTR of PVX and the BaMV replicase complex. The mutant rDE transcript exhibited a stronger affinity (K_M_=209 nM) but a slower initiation rate that cause a lower V_max_/K_M_ than rABCDE in the interaction with the BaMV replicase complex.

**Table 1:** **Kinetic parameters of the *in vitro *BaMV RNA replication for the short transcript templates**

	**rABCDE**^**a**^	**rABCP**	**rP**	**rDE**
^b^K_M_ (nM)	302^e^	296	533	209
^c^V_max_ (min^-1^) (rel V_max_)	63 (1.00)	21 (0.33)	25 (0.40)	17 (0.25)
^d^V_max_/K_M_ (mM^-1^s^-1^) (rel V_max_/K_M_)	3.477 (1.00)	1.182 (0.34)	0.782 (0.22)	1.356 (0.34)

^a^The short transcripts used in the *in vitro* BaMV RNA replication are indicated in Figure 1.

^b^Apparent K_M_ is defined as the concentration of RNA molecules that permits half-maximal rate of reaction. It stands for how effectively the enzyme would bind the substrate.

^c^V_max_ is the maxim initial velocity that an enzyme can achieve. It stands for the rate at which a substrate will be converted to product once bound to the enzyme. The relative activity is shown in the bracket.

^d^V_max_/K_M_ stands for the overall specificity of the enzyme reaction.

^e^The parameters were calculated by creating a Lineweaver-Burk plot derived from the Michaelis-Menten equation (Figure 5).

## Discussion

The 3′ UTR of BaMV RNA was proven to be essential for viral RNA replication in protoplasts [14-16]. The chimera BaMV-S/ABCP failed to accumulate any viral products in protoplasts; however, the rABCP transcript exhibited a wild-type level of RNA synthesis in the *in vitro* replication assay (Figures 2 and 4). The results of the kinetic study demonstrated that the V_max_/K_M_ of rABCP was a three-fold lower than that of rABCDE. The RNA replication rate of this mutant in a cell may be too slow to compete out with the rate of viral RNA removal by the scavenging system such as the siRNA silencing pathway [22]. The catalytic constant in viral RNA synthesis was critical to establish the successful viral infection that the higher catalytic in transcription the higher the chances to survive in hosts [23,24]. Alternatively, minus-strand RNA synthesis may be normally executed *in vivo* as that shown in the *in vitro* replication assay, however, the progeny plus-strand RNA derived from these newly synthesized minus-strand templates contains the aberrant sequences (the chimera) in the 3′ UTR. These abnormal chimeric 3′ UTRs may cause the failure in post-transcription RNA maturation (such as polyadenylation) and result in immature progeny RNAs.

The long-distance interaction between the 3′ and 5′ end of RNA plays a vital role in the synthesis of positive-strand RNA, because the 3′ end sequence of BMV [25], the satellite RNA C associated with TCV [26], and mouse hepatitis virus [27,28] have all been demonstrated to be involved in regulating positive-strand RNA synthesis [29]. Furthermore, the sequence complementarities and direct RNA-RNA interaction between both ends of RNA were reported in the influenza virus [30-32] and vesicular stomatitis virus [33-35]. However, these types of interactions were not elucidated so far in BaMV.

Compared to rABCP (93%; Figure 4B), the reduction of RNA replication with rABCDP (51%) and rABCPE (38%) in an *in vitro* BaMV RNA replication assay may be attributed to the redundant potexviral-conserved hexamer motifs that are found in D domain of BaMV RNA and the 3′ UTR of PVX RNA. An alternative hypothesis is that the alteration of the distance between the ABC domain and the poly(A) region, which was demonstrated to be the initiation site of minus-strand RNA replication [19], may hinder the progression of RNA replication, and that rABCP and rABCDE may fold into a similar conformation. Footprinting analysis demonstrated that domains D and E are the target sites for the polymerase domain of BaMV ORF1 [6]. Therefore, the space between domain D and E is critical for the interaction between the RNA template and the RdRp complex. The differences between the kinetic parameters of rABCP (K_M_=295 nM) and rP (K_M_=533 nM) imply that the addition of the BaMV ABC domain enhanced the interaction between the BaMV RdRp complex and the 3′ UTR of PVX. This enhancement may be resulting from the binding of the ABC domain in the 3′ UTR with the helicase-like domain of the replicase encoded by BaMV [15]. The helicase-like domain in BMV played a role in RNA recruitment [36] that the disrupting or missing the ABC (the helicase binding region in BaMV) domain in the 3′ UTR of BaMV may also be failure in template recruitment. The rDE transcript exhibited a higher affinity with the replicase complex, but failed to replicate in protoplasts. The V_max_/K_M_ of rDE was calculated as 2.5-fold lower than that of rABCDE. A stronger binding may impede the movement of the enzyme complex on the RNA template, which may lead to an RNA replication rate too slow to overcome the threshold required to establish a successful infection.

## Conclusion

Among the various BaMV/PVX chimera mutants, the BaMV-S/PABCDE that contained the uninterrupted BaMV 3′ UTR is the only mutant that exhibited a wild-type level of viral product accumulation in protoplasts and plants. These results indicate that the integrity of the 3′ UTR is vital for BaMV RNA replication. Although the 3′ UTR of PVX could functionally replace the D and E domains in the 3′ UTR of BaMV *in vitro*, the three-fold less of the minus-strand RNA synthesis rate than that of wild type could be the main cause of the failure in accumulation *in vivo*. Overall, these results provide additional support to our previous studies because the specific sequences and structures within each domain of the 3′ UTR may be recognized by the various domains of the BaMV replicase [6,15].

## Methods

### Plasmids construction

The BaMV strain S-derived infectious cDNA clone, pBS2-8 (EMBL/GenBank accession no. AF018156; Liao and Hsu, unpublished) and pPVX/UTR containing the 3′ UTR of PVX RNA [12] were used for mutant construction. Six primers Ba3′P (5′CCCGAACCAACATCAGACTAACTACGTCTACATAACCGA3′), Ba/ABCP (5′GAAAGAAAGGTTTACACCTACGTCTACATAACCGA3′), Ba/ABCDP (5′GCCAGCAGAATAAAGACCCTACGTCTACATAACCGA3′), Ba/PABCDE (5′GAATAATATAAATACTAAACGTTGCATGAT3′), Ba/PDE (5′GTATGAATAATATAAATTTTACACGGACTCTGTT3′), and Ba/PE (5′GTATGAATAATATAAATATAAAGACCTTTT3′) in combination with the downstream primer *Sac*I-polyT (5′GAGCTCT_40_) were used to generate PCR products. Each of the 181- to 115-bp PCR fragments was used subsequently as the megaprimers together with an upstream primer, BaMV6122 (5′GCCAATGACCAGAAAGGGTTCAA3′), to generate the 478- to 452-bp DNA fragments through a second PCR using pBS2-8 as the template [37].

All the PCR products were cloned into the pGEM® -T Easy vector (Promega, Madison, WI, USA) and verified by DNA sequencing. The *Sap*I^6207^-to-*Sac*I (located 3′ to the insert) fragment was then subcloned into pBS2-8 to replace the corresponding *Sap*I-to-*Sac*I region. Because pBS2-8 has two *Sap*I sites, mutant constructs were done by three-fragment ligation and confirmed by DNA sequencing. These mutant plasmids were designated as pBaMV-S/P, -/ABCP, -/ABCDP, -/PABCDE, -/PDE, -/PE, and -/ABCPE, respectively (Figure 1).

To construct the mutant pBaMV-S/ABCDEP, the first PCR product (694 bp) generated with the template pBS2-8 and the primers BaMV5768(+) (5′GGCCTCAGTCTCGAAGC TTTCGA3′) and gg15T+PVX3T (5′TTTATTTGTATTATTCATACAATCAAATCAAACCA GAAAATACTATGAAACTGGGGTAGGCGTCGGTTATGTAGACGTAGGTTTTTTTTTTTTTTTGGA3′) was used as the template for the second PCR. The second PCR product (717 bp) amplified with primers BaMV5768(+) and SacI40TPVX(−) (5′GAGCTCTTTTTTT TTTTTTTTTTTTTTTTTTTTTTTTTTTTTTTTTATTTATATTATTC3′) was cloned to pGEM® -T Easy vector and sequenced. The insert in the vector was digested with *Hind*III and *Sac*I and replaced to that of pBS2-B.

### *In vitro* RNA transcription

Before *in vitro* transcription, all plasmids were linearized with *EcoICR*I (Promega). The reaction was carried out at 37°C for 2 h in a 50-μl reaction containing 100 U of T7 RNA polymerase, 40 mM Tris–HCl pH 8.0, 8 mM MgCl_2_, 2 mM spermidine, 10 mM DTT, 0.5 mM GTP, 1 mM ATP, 1 mM UTP, 1 mM CTP, 2 mM m^7^GpppG cap analogue, and 5 μg of the linearized template. Subsequently, the reaction was supplemented with 5 U of RNase-free DNase I and incubated at 30°C for 20 min to degrade the DNA template. Transcription reactions were brought up to 200 μl with TE and extracted with an equal volume of phenol/chloroform. RNA was precipitated with ammonium acetate and ethanol. The pellet was dissolved in 300 μl of water, and 200 μl of 7.5 M ammonium acetate was added. The mixture was incubated at −80°C for 20 min, thawed at room temperature, and centrifuged at 4°C for 20 min. RNA transcripts were washed with 70% ethanol, dried, dissolved in 20 μl of water, and quantified by running on a 1% agarose gel with control markers.

### Protoplast and plant inoculation

Four grams of sliced *N. benthamiana* leaves were digested with 25 ml of enzyme solution containing 0.55 M Mannitol-MES, pH 5.7, 0.1% bovine serum albumin, 0.6 mg/ml macerase pectinase (Calbiochem, La Jolla, CA), and 12 mg /ml cellulase (Yakult Pharmaceutical,Tokyo, Japan) at 25°C for overnight. The mesophyll cells were spun down at 300 rpm (KUBOTA KS-5000) for 7 min and suspended in 2 ml of 0.55 M Mannitol-MES buffer. Intact protoplasts were collected from the interphase above the 0.55 M sucrose cushion and washed with Mannitol-MES buffer at least two times. A total of 4 × 10^5^ cells were transfected with 5 μg of RNA transcripts with the help of polyethylenglycol. Finally, the transfected protoplasts were suspended in culture medium (1 μM CuSO_4_, 10 mM MgSO_4_, 1 μM KI, 0.2 mM KPO_4_, 10 mM KNO_3_ pH 6.5, 10 mM CaCl_2_, 0.03% cephaloridins, 0.001% gentamycin and 0.55 M Mannitol-MES) and incubated under a constant light at 25°C for 48 h.

*N. benthamiana* seedlings were grown under 16-h illumination at 28°C until plants had about four leaves. Five or 20 μg of the RNA transcripts or lysates of the inoculated protoplasts (about 10^5^ cells) were gently rubbed onto one leaf of each plant. The inoculated and systemically infected leaves were harvested 10 days after inoculation.

### Northern blotting analysis

The detection probe was a ^32^P-labelled 0.6-kb RNA transcript complementary to the 3′-end of BaMV RNA. The 20-μl *in vitro* transcription reaction contained 2 μg of template DNA (pBaMV-O/SB 2.6 linearized with *Hind*III [38]), 40 mM Tris–HCl pH 8.0, 8 mM MgCl_2_, 2 mM spermidine, 10 mM DTT, 3 mM ATP, 3 mM CTP, 3 mM GTP and 70 μCi[α-^32^p]UTP (Dupont-NEN, Boston, MA). Total RNA extracted from the inoculated protoplasts were incubated in a buffer containing 50% DMSO, 1 M glyoxal and 10 mM sodium phosphate pH 7.0 at 50°C for 1hr and resolved on a 1% agarose gel in 10 mM sodium phosphate buffer. The RNA was transferred onto a Zeta-Probe® blotting membrane (Bio-Rad Laboratories, Hercules, CA, USA) which was subsequently hybridized with the probe about one million-cpm at 60°C overnight [16]. After hybridization, the membrane was washed, and scanned with a phosphoimager (BAS-1500; Fujifilm, Tokyo, Japan).

### Western analysis

Total protein harvested from the RNA-inoculated protoplasts was separated in a 12% SDS-polyacrylamide gel and electroblotted onto a nitrocellulose membrane (PROTRAN® BA 85; Schleicher & Schuell, Germany). A primary rabbit against-BaMV coat protein polyclonal antiserum and a secondary fluorescence-labeled anti-rabbit IgG antibody (H&L) were used to detect BaMV coat protein on the membrane. Data analysis was carried out using the LI-COR Odyssey (LI-COR Biosciences, Lincoln, USA).

### *In vitro* translation and RNA stability assay

*In vitro* translation was performed in the TnT-coupled transcription/translation system (Promega, Carlsbad, CA, USA) according to the manufacturer. Briefly, the reaction was carried out in a 12.5 μl-reaction mixture containing 1 μg RNA in wheat germ extract supplemented with all amino acids except methionine. Proteins were labeled by the incorporation of 5 μCi of L-[^35^S] methionine (1,000 Ci/mmol; 10 mCi/ml; Perkin Elmer, Walthman, MA, USA) in the reaction. Incubations were performed at 30°C for 1 h and terminated by the addition of sample buffer. The translation products were analyzed by 10% SDS-polyacrylamide gel electrophoresis. Gels were dried, and analyzed by a phosphorimager (Fujifilm BAS 2500).

For RNA stability assay, RNAs were incubated with wheat germ extract supplemented with all amino acids. At various time points of post incubation, viral RNAs were isolated and analyzed by Northern blotting.

### Templates for *in vitro* replication assay

The RNA transcripts were generated directly from PCR-generated DNA templates using T7 RNA polymerase [39]. The specific primers, *EcoR*I-T7-6228 (5′GCGAATTCTAATACGACTCACTATAGGGCGTTGCATGATCG3′), T7-PVX (5′TAATACGACTCACTATAGGGTAACTACGTCTACAT3′), and the down stream primers, PVX40T (5′T_40_ATTTATATTATTCATAC3′) and 40TGG (5′T_40_GG3′), were used to synthesize the PCR products. The pPVX/UTR, pBaMV40A, pBaMV-S/ABCP, -/ABCDP, -/PDE, -/PE, -/PABCDE, -/ABCPE, and -/ABCDEP were employed as the template to generate rABCP, rABCDP, rPDE, rPE, rPABCDE, rABCPE, and rABCDEP, respectively. The RNA transcripts were gel-purified and quantified by spectrophotometry.

### *In vitro* BaMV RNA replication assay

For exogenous RNA template activity assay, the detergent-solubilized RdRp preparation [13] was treated with 20 μg/ml micrococcal nuclease (Pharmacia Biotech) in the presence of 2 mM CaCl_2_ at 30°C for 30 min and the treatment was stopped by adding 25 mM EGTA. The 50-μl BaMV RNA replication reaction contained 25 μl of the RdRp preparation, 10 mM DTT, 3 mM MgCl_2,_ 2 mM of each ATP, CTP, and GTP, 2 μM UTP, 2 μM (α-^32^P) UTP (3000 Ci/mmol; Dupont-NEN), 4.8 mg/ml bentonite, and 1 μg of RNA template. Reaction was incubated at 30°C for 1 hr and stopped by phenol/chloroform extraction, followed by ethanol precipitation. The pellet was dissolved in 5 μl water and treated with RNase A (32 μg/ml) and T1 (1.6 μg/ml) at 30°C for 30 min in RNase protection buffer (10 mM Tris–HCl, pH 7.5, 5 mM EDTA, 200 mM NaCl and 100 mM KCl) to collect only double-stranded RNA. After incubation at 37°C for 30 min with 66 mg/ml proteinase K [40], RNA was extracted with phenol/chloroform and precipitated in ethanol. The reaction products were resolved by gel electrophoresis and detected by using a Bio-Imaging analyzer BAS-1500. The kinetic parameters of the rABCDE, rABCP, rP, and rDE templates were determined by taking measurements at six time points (10, 20, 40, 60, 90, and 120 min) in the presence various template concentrations (30 to 1500 nM). The initial rates were plotted onto a Michaelis-Menten graph and the K_M_ and V_max_ were calculated by the double-reciprocal Lineweaver-Burk plot.

## Competing interests

The authors declare that they have no competing interests.

## Authors’ contributions

IHC and CHC performed most of the plasmids construction, *in vitro* BaMV replication assay, and protoplast and plant inoculation. JWL contributed to this work with BaMV replicase complex purification. YHH supplied the antibody and partially subsidized the study. CHT designed the experiments, analyzed data, and contributed to finalizing the manuscript. All authors read and approved the final manuscript.
